# Atopic Dermatitis: Natural History, Diagnosis, and Treatment

**DOI:** 10.1155/2014/354250

**Published:** 2014-04-02

**Authors:** Simon Francis Thomsen

**Affiliations:** Department of Dermatology, Bispebjerg Hospital, Bispebjerg Bakke 23, 2400 Copenhagen NV, Denmark

## Abstract

Atopic dermatitis is an inflammatory skin disease with early onset and with a lifetime prevalence of approximately 20%. The aetiology of atopic dermatitis is unknown, but the recent discovery of filaggrin mutations holds promise that the progression of atopic dermatitis to asthma in later childhood may be halted. Atopic dermatitis is not always easily manageable and every physician should be familiar with the fundamental aspects of treatment. This paper gives an overview of the natural history, clinical features, and treatment of atopic dermatitis.

## 1. Definition 

Atopic dermatitis is a common, chronic, relapsing, inflammatory skin disease that primarily affects young children.* Atopy* is defined as an inherited tendency to produce immunoglobulin E (IgE) antibodies in response to minute amounts of common environmental proteins such as pollen, house dust mites, and food allergens. Dermatitis derives from the Greek “derma,” which means* skin,* and “itis,” which means* inflammation*. Dermatitis and* eczema* are often used synonymously, although the term eczema is sometimes reserved for the acute manifestation of the disease (from Greek, ekzema,* to boil over*); here, no distinction is made. Over the years, many other names have been proposed for the disease, for instance, prurigo Besnier (*Besnier's itch*), named after the French dermatologist Ernest Besnier (1831–1909). Allergic sensitization and elevated immunoglobulin E (IgE) are present in only about half of all patients with the disease, and therefore* atopic dermatitis* is not a definitive term.

## 2. Epidemiology

Atopic dermatitis affects about one-fifth of all individuals during their lifetime, but the prevalence of the disease varies greatly throughout the world [1]. In several so-called industrialised countries, the prevalence increased substantially between 1950 and 2000 so much that many refer to as the “allergic epidemic.” However, current indications point to eczema symptoms having levelled off or even having decreased in some countries with a formerly very high prevalence, such as the United Kingdom and New Zealand. This indicates that the allergic disease epidemic is not increasing continually worldwide. Nevertheless, atopic dermatitis remains a serious health concern, and in many countries, particularly in the developing world, the disease is still very much on the rise.

### 2.1. Natural History

Around 50% of all those with atopic dermatitis develop symptoms within their first year of life, and probably as many as 95% experience an onset below five years of age [2]. Around 75% with childhood onset of the disease have a spontaneous remission before adolescence, whereas the remaining 25% continue to have eczema into adulthood or experience a relapse of symptoms after some symptom-free years. Many with adult-onset atopic dermatitis or atopic dermatitis relapsing in adulthood develop hand eczema as the main manifestation. In some patients, this constitutes a serious concern as it may affect their choice of career or employment and in some cases it may even lead to an early exit from the labour market.

Around 50–75% of all children with early-onset atopic dermatitis are sensitized to one or more allergens, such as food allergens, house dust mites, or pets, whereas those with late-onset atopic dermatitis are less often sensitized [3]. However, intake of foods or exposure to airborne allergens is rarely the cause of exacerbations in atopic dermatitis; many patients with the disease are sensitized to foods without this playing a role in eczema activity. Atopic dermatitis, particularly severe disease, in a child heralds other atopic diseases. A child with moderate to severe atopic dermatitis may have as much as 50% risk of developing asthma and 75% risk of developing hay fever [4].

### 2.2. Risk Factors

The risk of developing atopic dermatitis is much higher in those whose family members are affected. For example, the concordance rate of atopic dermatitis in monozygotic twins is around 75%, meaning that the risk of the disease in the twin sibling is 75% if the cotwin is affected [5]. In contrast, the risk in dizygotic twins is only 30%. This shows that genetic factors play a role in the susceptibility to atopic dermatitis. However, as there is not complete concordance between monozygotic twins, who share all their genes, environmental and developmental factors must play a role too. As such, atopic dermatitis is a complex genetic disease arising from several gene-gene and gene-environment interactions.

#### 2.2.1. Genetics

Many genes have been associated with atopic dermatitis, particularly genes encoding epidermal structural proteins and genes encoding key elements of the immune system. A recent and interesting genetic discovery is the documented strong association between atopic dermatitis and mutations in the* filaggrin* gene, positioned on chromosome 1 [6]. The* filaggrin* gene is the strongest known genetic risk factor for atopic dermatitis. Around 10% of people from western populations carry mutations in the* filaggrin* gene, whereas around 50% of all patients with atopic dermatitis carry such mutations.* Filaggrin* gene mutations give rise to functional impairments in the filaggrin protein and thereby disrupt the skin barrier. The clinical expression of such impairments is dry skin with fissures and a higher risk of eczema. Not all patients with atopic dermatitis have these mutations and other genetic variants have also been incriminated [7]. It is the combined action of all these genetic variants along with environmental and developmental risk factors that cause atopic dermatitis.

#### 2.2.2. Environment

Although many different environmental risk factors have been considered potentially causative for atopic dermatitis, only a few are consistently accepted. For example, there is substantial evidence that our western lifestyle leads to some of the reported increase in eczema occurrence over the past years although this has not pointed to specific environmental risk factors or has translated directly into functional preventive measures [8]. Many advocate the* hygiene hypothesis* when explaining the rapid increase in eczema prevalence [9]. This hypothesis states that the decrease in early childhood exposure to* prototypical* infections, such as hepatitis A and tuberculosis, has increased susceptibility to atopic diseases [10]. The hypothesis is supported by the observations that the youngest among siblings has the lowest risk of atopic dermatitis and that children who grow up in a traditional farming environment where they are exposed to a variety of microflora, for instance, from unpasteurized cow's milk, livestock, and livestock quarters, are protected to some extent against developing the disease and against allergic diseases in general [11]. In contrast, disease development is probably positively correlated with duration of breastfeeding [12], whereas several studies have linked a high social position of the parents to an increased risk of atopic dermatitis in the child [13]. Although such observations are not easy to interpret, they may also lend support to the hygiene hypothesis or at least to the generally accepted theory that eczema occurs in genetically susceptible individuals who are exposed to a certain disadvantageous environment.

## 3. Pathophysiology

Two main hypotheses have been proposed to explain the inflammatory lesions in atopic dermatitis. The first hypothesis concerns an imbalance of the adaptive immune system; the second hypothesis concerns a defective skin barrier. Although these two hypotheses are not thought to be mutually exclusive, they may complement each other.

### 3.1. Immunological Hypothesis

The theory of immunological imbalance argues that atopic dermatitis results from an imbalance of T cells, particularly T helper cell types 1, 2, 17, and 22 and also regulatory T cells [14]. In the allergic (atopic dermatitis) state—particularly in acute eczema—the Th2 differentiation of naive CD4+ T cells predominates. This causes an increased production of interleukins, primarily IL-4, IL-5, and IL-13, which then leads to an increased level of IgE, and the Th1 differentiation is correspondingly inhibited.

### 3.2. The Skin Barrier Hypothesis

The theory of skin barrier defects is more recent and has its origin in the observation that individuals with mutations in the* filaggrin* gene are at increased risk of developing atopic dermatitis [6]. The* filaggrin* gene encodes structural proteins in the stratum corneum and stratum granulosum that help bind the keratinocytes together. This maintains the intact skin barrier and the hydrated stratum corneum. With gene defects, less filaggrin is produced, leading to skin barrier dysfunction and transepidermal water loss, which causes eczema. There is evidence to suggest that the impaired skin barrier, which results in dry skin, leads to increased penetration of allergens into the skin, resulting in allergic sensitization, asthma, and hay fever [15]. Preventing dry skin and active eczema early in life via application of emollients may constitute a target of primary prevention of progression of eczema into allergic airways disease.

## 4. Histopathology

A skin biopsy taken from a site with acute atopic eczema is characterised by intercellular oedema, perivascular infiltrates primarily of lymphocytes, and retention of the nuclei of the keratinocytes as they ascend into the stratum corneum—so-called parakeratosis. Chronic eczema is dominated by a thickened stratum corneum, so-called hyperkeratosis, a thickened stratum spinosum (acanthosis), but sparse lymphocytic infiltrates.

## 5. Diagnosis and Clinical Presentation

The appearance of the individual skin lesion in atopic dermatitis does not differ from other eczemas such as contact eczema. In its acute form, eczema is characterised by a lively red infiltrate with oedema, vesicles, oozing, and crusting; lichenification, excoriations, papules, and nodules dominate the subacute and chronic form. Accordingly, the diagnostic approach builds upon other characteristics such as the distribution of the eczema as well as associated features of the patient. The typical patient with atopic dermatitis is a person with:

 
*an early onset of itchy eczema localised at typical sites such as the flexures of the elbows and knees in an atopic patient or in a person with a familial predisposition to atopic disease.*

The most widely used diagnostic criteria for atopic dermatitis were developed by Hanifin and Rajka in 1980 and were later revised by the American Academy of Dermatology (Table 1) [16].

This set of criteria is primarily useful in clinical practice; another set of diagnostic questions widely used in epidemiological research was developed by the UK Working Party in 1994 (Table 2) [17].

The severity of eczema can be graded according to several scoring systems such as SCORAD [18] and EASI [19].

### 5.1. Typical Manifestations

Although this description fits many with the disease, the clinical presentation of atopic dermatitis is often more elaborate with a large variation in the morphology and distribution of the eczema combined with various other features. However, many patients with atopic dermatitis have a general tendency to present with dry skin (xerosis) due to the low water content and an excessive water loss through the epidermis. The skin is pale because of increased tension in the dermal capillaries and the ability to sweat is reduced. There is an increased cholinergic response to scratch, so-called white dermographism or* skin-writing, *resulting in hives at the affected site. The palms of the hands and feet may show hyperlinearity, and the individuals' hair is dry and fragile. Often, there is a double skinfold underneath the inferior eyelid (Dennie-Morgan fold) that becomes exaggerated in times of increased disease activity. The eye surroundings may be darkened due to postinflammatory hyperpigmentation.

Atopic dermatitis can be grouped into three clinical stages, although these may be difficult to reproduce in the individual patient [2].

#### 5.1.1. Atopic Dermatitis of Infancy

Infants experience eczema that is often localised to the face, scalp, and extensor aspects of the arms and legs, but it can also be widespread. The lesions are characterised by erythema, papules, vesicles, excoriations, oozing, and formation of crusts.

#### 5.1.2. Atopic Dermatitis of Childhood

In toddlers and older children, the eczema lesions tend to shift location so that they are often confined to the flexures of the elbows and knees as well as the wrists and ankles, although it can occur at any site. In general, the eczema becomes drier and lichenified with excoriations, papules, and nodules.

#### 5.1.3. Atopic Dermatitis of Adolescence and Adulthood

In adult patients, the lesions frequently localise to the face and neck,* head-and-neck dermatitis*, and a considerable portion of patients, around 30%, develop atopic hand eczema, which may interfere with workplace activities.

### 5.2. Special Manifestations

Some patients may present with several other common, benign skin conditions, for example,* pityriasis alba*, which is a condition characterised by dry, pale patches on the face and upper arms, and* keratosis pilaris*, which manifests as small, rough keratotic papules particularly on the upper arms and thighs. Atopic winter feet—*dermatitis plantaris sicca*—a condition usually seen in school-aged children is characterised by symmetric eczema on the weight bearing areas of the soles of the feet. Earlobe eczema, eczema of the nipple, and eczema around the margins of the mouth (cheilitis) can be particularly troublesome and often involve infection with staphylococci. Keratoconus and cataracts sometimes complicate atopic dermatitis.

### 5.3. Aggravating Factors

In many patients, atopic dermatitis takes a chronic, relapsing course when it is not possible to predict periods of activity or pinpoint aggravating factors. However, several exposures are well known for aggravating eczema and should be avoided. A large number of patients are sensitive to woollen clothing, which aggravates itching and discomfort. Hot water may also exacerbate itching, and long baths should be avoided. Several infections, notably staphylococci, are frequent causes of exacerbations as various foods are, particularly in cases where a patient is sensitized to the food. Food avoidance should be advocated only if a patient has documented allergy to a suspected food and not on the basis of asymptomatic sensitization alone. Another phenomenon that can lead to the eczema worsening is contact urticaria, which is a reaction following skin exposure to a food, for example, citrus fruits or tomatoes. The skin around the mouth is often the site of such a reaction. Lastly, many patients report that stressful living aggravates their eczema.

### 5.4. Differential Diagnoses

Several diseases present with a skin rash that resembles atopic dermatitis. However, careful evaluation of the morphology and localization of the rash combined with information about the individual patient usually leads to a diagnosis. Diseases that sometimes resemble atopic dermatitis are scabies, seborrheic dermatitis, and contact dermatitis.

### 5.5. Complications

Several microorganisms, such as bacteria, viruses, and fungi, can complicate the eczema (causing superinfections). The skin of the patient with atopic dermatitis is often colonised with* Staphylococcus aureus*, particularly when the eczema is not well controlled. The mere presence of such bacteria does not require antibiotic treatment. However, if staphylococci become invasive, oozing crusted lesions—*impetigo*—can be the result, which indicates the need for topical or, preferably, oral antibiotics [20]. Some advocate skin washing with antiseptic remedies, such as chlorhexidine, as this lowers the number of bacteria on the skin; however, chlorhexidine can lead to secondary sensitization. Due to deficiencies in the production of antimicrobial peptides in the skin, patients with atopic dermatitis also have a greater risk of several viral infections, for example, molluscum contagiosum, caused by a pox virus, which gives small, umbilicated, dome-shaped, pearly coloured papules. Another typical superinfection of the skin in atopic dermatitis patients is herpes virus. If such a herpes infection spreads, it can cause* eczema herpeticum*, which is a widespread vesicular eruption, typically localised to the face, scalp, and upper chest. Eczema herpeticum requires systemic antiviral treatment.

## 6. Treatment

Atopic dermatitis is not curable, and many patients will experience a chronic course of the disease. Accordingly, the treatment of atopic dermatitis aims to [21]

1. minimise the number of exacerbations of the disease, so-called* flares*,
2. reduce the duration and degree of the flare, if flare occurs.

The first aim relates primarily to prevention; the second aim relates to treatment. Prevention is best attained by trying to reduce the dryness of the skin, primarily via daily use of skin moisturising creams or* emollients* along with avoidance of specific and unspecific irritants such as allergens and noncotton clothing. When dryness is reduced, the desire to scratch will lessen and the risk of skin infection will decrease. Avoiding long, hot baths further prevents skin dryness, but when a bath is taken, an emollient should be applied directly after it to secure a moist epidermis and augment the skin barrier function. Reducing the flare is warranted when actual eczema occurs or when mild intermittent eczema worsens. Management of an eczema exacerbation requires medical treatment often in the form of corticosteroid creams. In addition to topical treatment, severe acute or chronic eczema often requires systemic immunosuppressant drugs or phototherapy (ultraviolet, UV light).

### 6.1. Emollients: Maintaining an Intact Skin Barrier

The use of emollients in the management of atopic dermatitis is pivotal. They should be applied several times a day, and a systematic use has been shown to reduce the need for corticosteroid creams [22, 23]. The main reason for intensive use of an emollient is its ability to increase the hydration of the epidermis, mainly by reducing the evaporation, as it acts as an occlusive layer on the top of the skin. As such, emollients have no direct effect on the course of the eczema. However, the appearance of the skin is enhanced and itching is reduced. Other moisturizers have more complex modes of action as they act by restoring the structural (lipid) components of the outer skin layers, thereby reducing cracks and fissures. Others act by attracting water molecules from the air in order to moisturize the skin. The choice of emollient depends on the individual patient. It is generally recommended that a thick (with a high fat content) cream or ointment is used for the driest skin, whereas creams and lotions with a higher water content are used only for very mild eczema. Such creams must be applied several times a day because of their rapid absorption into the skin. It is important to recommend an emollient without perfume or other potential allergens as they may provoke secondary allergic sensitization. Those with chronic, dry eczema benefit from tar preparations in the form of creams and occlusive bandages.

### 6.2. Topical Corticosteroids

Topical corticosteroids are the mainstay of the treatment for moderate to severe atopic dermatitis, both in children and adults. Corticosteroids are hierarchically grouped into different classes based on their vasoconstrictory abilities. For ease, four classes are considered: mild, moderate, strong, and very strong preparations (Table 3).

#### 6.2.1. How Should Corticosteroids Be Applied?

Most patients benefit from treatment with mild to moderate corticosteroid preparations, whereas only a small subset—those with severe disease—needs potent preparations; very strong preparations are rarely needed. Mild and moderate corticosteroid creams are reserved for children, while adults can be treated with stronger preparations. Mild and moderate corticosteroids should be used chiefly for treating eczema on body sites where the skin is thin, notably in the face, axillae, groins, and anogenital area, whereas strong corticosteroids should be used for treating eczema on the rest of the body. Unlike medications used for treating asthma and allergic rhinitis, creams for atopic dermatitis are not prepared with a fixed amount of drug release per round of usage. Instead, the “rule of the fingertip unit (FTU)” must be applied. A fingertip unit is the amount of cream or ointment squeezed from a standard tube along an adult's fingertip—a fingertip is from the very end of the finger to the distal crease in the finger. One FTU is sufficient to treat an area of skin twice the size of the flat of an adult hand with the fingers together (Table 4).

As one FTU equals roughly 0.5 g cream, the amount needed to adequately treat an entire adult body surface once is 20 g, whereas a 1-2-year-old child, for instance, requires about 7 g.

#### 6.2.2. Proactive and Reactive Treatment

Corticosteroid creams are used both for treating acute flares of atopic dermatitis and for maintenance therapy; that is, prevention of disease relapses when the acute flare is under control. For treating acute flares, one daily application is recommended of the cream with the lowest potency deemed sufficient to clear the eczema within 1-2 weeks [24]. When the eczema flare is well controlled, that is, when the rash is quiescent and particularly when the itch has subsided substantially, use of the corticosteroid cream should be tapered off to two to three weekly applications for an additional 1-2 weeks. Another tapering approach is to use a lower potency cream daily for 1-2 weeks. However, patients may find this approach slightly more difficult to manage. In theory, treatment could be discontinued at the end of the tapering period if the flare is sufficiently under control, but in many patients the eczema relapses, and an additional round of treatment is required. If this is the case, it is preferable to continue the maintenance treatment, applying the corticosteroid cream two to three times weekly on those sites—for instance, the elbow creases—likely to become active again if treatment is discontinued. This strategy is called the* proactive* treatment strategy, as compared with the* reactive* strategy, which recommends intermittent use of the corticosteroid preparation according to the activity of the eczema. The proactive treatment strategy is being increasingly advocated because the overall quantity of corticosteroid cream used is smaller than that used with the reactive treatment strategy; additionally, the risk of an exacerbation of the eczema is smaller when using the proactive treatment strategy.

#### 6.2.3. Side Effects

Patients and physicians alike fear the cutaneous and systemic side effects from using topical corticosteroids. However, although topical corticosteroids can cause thinning of the skin, teleangiectasies, and stretch marks, when used properly, the risk of side effects is very small. It is essential that physicians try to reassure parents of atopic children and the patients themselves and explain that this fear of side effects should not inhibit the use of corticosteroids since insufficient use can cause worsening of the eczema. Including the patient (and the parents) in the treatment plan is paramount. Rather than dictating what is best for the child, physicians should discuss the parents' concerns in order to avoid disrupting the physician-patient-parent relationship, which would ultimately lead to complications for the child.

### 6.3. Calcineurin Inhibitors

Pimecrolimus cream and tacrolimus ointment—also termed topical calcineurin inhibitors—are newer formulations used both for the treatment of acute flares and for maintenance therapy of atopic dermatitis [25]. Pimecrolimus has the potency of a mild corticosteroid cream, whereas tacrolimus corresponds to a moderate to strong topical corticosteroid. The side effects of corticosteroids, such as thinning of the skin, are not seen with topical calcineurin inhibitors, and this allows daily treatment for longer periods. Topical calcineurin inhibitors can also be used in the proactive treatment strategy.

### 6.4. Phototherapy

Widespread eczema benefits from treatment with UV light. Narrowband UVB light is particularly suitable for treating adults with recalcitrant eczema. Broadband UVA light and a combination of UVA light and the photosensitizing drug* psoralene* can also be used to treat severe recalcitrant eczema. Difficult-to-treat atopic dermatitis often clears with 1-2 months' phototherapy three to five times a week, preferably combined with topical corticosteroids. Nevertheless, as phototherapy causes premature aging of the skin and increases the risk of skin cancer in the long run, it should be prescribed with caution.

### 6.5. Systemic Immunosuppressant Treatments

Short-term tapered treatment with oral corticosteroids is recommended for acute flares of severe, widespread atopic dermatitis, preferably in combination with topical corticosteroids. As* Staphylococcus* infections often trigger such flares, oral antibiotics should be prescribed simultaneously. Due to the risk of side effects, continuing treatment with oral corticosteroids is not recommended. Instead, tapering should be done while introducing a second immunosuppressant drug, for example, azathioprine, methotrexate, or cyclosporine A, for very severe, chronic, relapsing atopic dermatitis [26]. Such treatment should be administered from specialised clinics or, preferably, from hospital dermatology departments.

### 6.6. Other Medications

Specific immunotherapy in patients with atopic dermatitis mainly has an effect on upper airway symptoms if the patient has concomitant allergic rhinitis, whereas the effect on the activity of the eczema is negligible.

Oral antihistamines are recommended for itching but have no effect on the activity of the eczema. Nonsedating antihistamines should be used, but when night-time itching interferes with sleep, sedating antihistamines are recommended.

## Figures and Tables

**Table 1:** Diagnostic criteria for atopic dermatitis.

Essential features	
Itch	
Eczema with typical morphology and age-specific pattern	
Important features	
Early age of onset	
Atopy (personal or family history)	
Dry skin	
Associated features	
Atypical vascular response (i.e., facial pallor, white dermographism)	
Keratosis pilaris, palmar hyperlinearity, ichthyosis	
Ocular and periorbital changes	
Other regional findings (e.g., perioral and periauricular lesions)	
Perifollicular accentuation, lichenification, and excoriations	

Modified from American Academy of Dermatology [16].

**Table 2:** Therapeutic approaches to atopic dermatitis.

Topical treatments	
Corticosteroids	
Calcineurin inhibitors	
Phototherapy	
Ultraviolet light A (UVA)	
Ultraviolet light B (UVB)	
Ultraviolet light A + Psoralene (PUVA)	
Systemic treatments	
Oral corticosteroids	
Azathioprine	
Cyclosporine A	
Methotrexate	

**Table 3:** Topical corticosteroids.

Mild (Class I)	
Hydrocortisone	
Moderate (Class II)	
Hydrocortison-17-butyrate	
Clobetason-17-butyrate	
Strong (Class III)	
Betamethason-17-valerate	
Fluticasone propionate	
Betamethasone	
Mometasonfuroate	
Desoximethasone	
Fluocinonide	
Fluocinolonacetonide	
Very strong (Class IV)	
Clobetasol propionate	

**Table 4:** Fingertip unit.

Area that needs treatment	FTUs (adults)	FTUs (children 1-2 years)
Face and neck	2.5	1.5
One hand and fingers	1	0.5
One arm, hand, and fingers	4	1.5
Chest and abdomen	7	2
Back and buttocks	7	3
One leg and foot	8	2
